# Nanophotosensitizers Composed of Phenyl Boronic Acid Pinacol Ester-Conjugated Chitosan Oligosaccharide via Thioketal Linker for Reactive Oxygen Species-Sensitive Delivery of Chlorin e6 against Oral Cancer Cells

**DOI:** 10.3390/ma15207057

**Published:** 2022-10-11

**Authors:** Sung-Ok Hong, Min-Suk Kook, Young-IL Jeong, Min-Ju Park, Seong-Won Yang, Byung-Hoon Kim

**Affiliations:** 1 Department of Oral and Maxillofacial Surgery, School of Dentistry, Kyung Hee University, Seoul 02447, Korea; 2 Department of Oral and Maxillofacial Surgery, Kyung Hee University Dental Hospital at Gangdong, Seoul 05278, Korea; 3 Department of Maxillofacial Oral Surgery, School of Dentistry, Chonnam National University, Gwangju 61186, Korea; 4 Department of Dental Materials, College of Dentistry, Chosun University, Gwangju 61452, Korea; 5 Department of Ophthalmology, College of Medicine, Chosun University, Gwangju 61453, Korea

**Keywords:** photodynamic therapy, oral cancers, nanophotosensitizers, reactive oxygen species, tumor-targeting

## Abstract

Chlorin E6 (Ce6)-incorporated nanophotosensitizers were fabricated for application in photodynamic therapy (PDT) of oral cancer cells. For this purpose, chitosan oligosaccharide (COS) was conjugated with hydrophobic and reactive oxygen species (ROS)-sensitive moieties, such as phenyl boronic acid pinacol ester (PBAP) via a thioketal linker (COSthPBAP). ThdCOOH was conjugated with PBAP to produce ThdCOOH-PBAP conjugates and then attached to amine groups of COS to produce a COSthPBAP copolymer. Ce6-incorporated nanophotosensitizers using the COSthPBAP copolymer were fabricated through the nanoprecipitation and dialysis methods. The Ce6-incorporated COSthPBAP nanophotosensitizers had a small diameter of less than 200 nm with a mono-modal distribution pattern. However, it became a multimodal and/or irregular distribution pattern when H_2_O_2_ was added. In a morphological observation using TEM, the nanophotosensitizers were disintegrated by the addition of H_2_O_2_, indicating that the COSthPBAP nanophotosensitizers had ROS sensitivity. In addition, the Ce6 release rate from the COSthPBAP nanophotosensitizers accelerated in the presence of H_2_O_2_. The SO generation was also higher in the nanophotosensitizers than in the free Ce6. Furthermore, the COSthPBAP nanophotosensitizers showed a higher intracellular Ce6 uptake ratio and ROS generation in all types of oral cancer cells. They efficiently inhibited the viability of oral cancer cells under light irradiation, but they did not significantly affect the viability of either normal cells or cancer cells in the absence of light irradiation. The COSthPBAP nanophotosensitizers showed a tumor-specific delivery capacity and fluorescence imaging of KB tumors in an in vivo animal tumor imaging study. We suggest that COSthPBAP nanophotosensitizers are promising candidates for the imaging and treatment of oral cancers.

## 1. Introduction

Most types of oral cancers are squamous cell carcinoma in the oral cavity, and they are easy to find at an early stage compared to systemic cancers [1]. Even though oral cancers are able to be cured at an early stage, they are frequently diagnosed at an advanced stage, with metastasis to other regions. Thus, at this stage, it makes treatment difficult in spite of easy accessibility [2,3]. Furthermore, oral cancers at an advanced stage lead to high recurrence rates and low response rates against single or combined treatments with these regimens [4,5,6]. For the treatment of oral cancer, various treatment regimens, such as surgery, immunotherapy, chemotherapy, and radiotherapy have been attempted [5,6,7,8]. Despite the progress of these treatment options, the 5-year survival rate of patients with advanced-stage oral cancers has decreased to less than 20% [9]. Even though chemotherapy or radiotherapy still remains the most common treatment option for oral cancer, their side effects are also problematic [10,11,12]. Specifically, the low selectivity of chemotherapeutic agents against oral cancer cells is regarded as the main inconvenience of chemotherapy [10]. Side effects such as oral mucositis after chemo- or radiotherapy decrease the life quality of patients [12,13]. To improve treatment efficacy and reduce side effects, novel treatment options for oral cancers have to be developed.

Photodynamic therapy (PDT) has been extensively investigated for the treatment of malignant disorders since it is composed of non-toxic components, such as light, oxygen, and photosensitizers [14,15,16,17]. In other words, it can be considered a safe treatment option with minimal side effects for cancer patients because it produces an excess amount of reactive oxygen in the field of light irradiation and low cytotoxic effects against the surrounding normal tissues. However, the application of PDT for cancer is limited to squamous or epithelial carcinoma because the depth of light penetration through the biological interface is limited to less than 15 mm, which is the limit of the light irradiation depth for the production of ROS by photosensitizers [17,18,19]. For these reasons, PDT for cancer treatment is regarded as an ideal candidate for oral cancers, including gingival cancers, which take place in the oral cavity, and visible light is easily accessed in these regions [20,21,22]. Jin et al. reported that PDT has efficacy in decreasing oral cancer with an overall complete response in their systemic review study [21]. Khan et al. reported that 5-amino levulinic acid (5-ALA)-based PDT can be used to control and monitor early oral cancer using smartphones [22]. From these points of view, PDT has been employed to control early and/or advanced stages of oral cancers [22,23,24]. However, a resistance problem in oral cancer was reported in traditional photosensitizers, such as 5-ALA [25]. Traditional photosensitizers spread out throughout the whole body by systemic and/or local administration since they have no specificity against tumor cells [14,15,16,25,26]. These problems induce adverse side effects, such as urticarial reaction, dermatitis, hyperpigmentation, etc. [27,28,29]. Furthermore, photosensitizers can remain in normal tissue for a long time. These problems require the blocking of sunlight to prevent phototoxicity [26,28,29].

Nanomedicine has been investigated as a diagnosis for the therapeutic purpose of cancer because it has targeting potential to specific sites of the body [30,31,32,33]. Since nanoparticles have unique properties, such as a small diameter and a huge surface area, they can be modified to be sensitive to the physiological status of tumors and to release relatively concentrated anticancer drugs in the tumor tissue [30,31,32,33]. Liu et al. reported that indocyanine green (ICG)-loaded nanoparticles show higher cellular uptake and stronger PDT efficacy against breast cancer cells, with no perceptible toxicity against normal mice [33]. Liang et al. reported that titanium dioxide nanoparticles decorated with folic acid depress HeLa tumor xenografts at a low dose of photosensitizers compared to free photosensitizers through a targeted delivery against tumor tissue [34]. Tumor microenvironments are quite different compared to normal tissues, i.e., the abnormalities of tumor microenvironments include an acidic pH, increased redox potential, and higher metabolic activity than normal cells or tissues [35,36,37]. Among these, the increased redox potential of tumor microenvironments can be applied to a target tissue of a nanoparticle-based drug delivery system [37]. Specifically, PDT induces an ROS-rich environment in the tumor tissue through light irradiation in the specific region. Nanoparticles can be modified to be sensitive against elevated redox potential in tumor tissues and then emphasize PDT efficacy [38,39]. Ruan et al. reported that redox-sensitive nanoparticles efficiently delivered the photosensitizers and then enabled the image-guided PDT of tumors [39].

In this study, we synthesized chitosan oligosaccharide (COS) conjugated with phenyl boronic acid pinacol ester via thioketal linker (COSthPBAP) and then fabricated chlorin e6 (Ce6)-incorporated nanophotosensitizers for the ROS-sensitive PDT of oral cancer cells. PBAP moieties may act as hydrophobic moieties, and then the COSthPBAP can be formed into nanoparticles in the aqueous environment. Furthermore, Ce6-incorporated COSthPBAP nanophotosensitizers can be fully degraded in an ROS-sensitive manner since PBAP and the thioketal linker have ROS-sensitive degradability [40,41]. We studied the ROS sensitivity, intracellular delivery capacity, and antitumor activity of Ce6-incorporated COSthPBAP nanophotosensitizers in vitro and in vivo.

## 2. Materials and Methods

### 2.1. Chemicals

Chitosan oligosaccharide (COS), triethylamine (TEA), N-(3-dimethylaminopropyl)-N’-ethylcarbodiimide hydrochloride (EDAC), N-hydroxy succinimide (NHS), and dimethyl sulfoxide (DMSO) were purchased from Tokyo Chemical Industry (TCI) Co., LTD. (Tokyo, Japan). Chlorin e6 (Ce6) was obtained from Frontier Sci. Co. (Logan, UT, USA). Thioketal dicarboxylic acid (ThdCOOH) was purchased from RuixiBiotech Co. Ltd. (Xi’an, China). Hydrogen peroxide (H_2_O_2_), phosphotungstic acid, 4-(aminomethyl) phenylboronic acid pinacol ester hydrochloride (PBAP), 2′,7′-dichlorofluorescin diacetate (DCFH-DA), 3-(4,5-dimethyl2-thiazolyl)-2, 5-diphenyl-2H-tetrazolium bromide (MTT), and 2,2,2-tribromoethanol (avertin) were purchased from Sigma Aldrich Chem. Co. (St. Louis, MO, USA). Dialysis membranes with molecular weight cutoffs (MWCO) of 1000 or 2000 Da were purchased from Spectrum Labs., Inc. (Rancho Dominguez, CA, USA). 2,2,2-tribromoethanol (Avertin) and tert-amyl alcohol were purchased from Sigma Aldrich Chem. Co. (St. Louis, MO, USA).

### 2.2. Synthesis of COSthPBAP Conjugates

To synthesize COSthPBAP conjugates, PBAP was primarily conjugated with one end of the carboxylic acid of ThdCOOH as follows: 224 mg of ThdCOOH was dissolved in 5 mL of DMSO with 192 mg of EDAC and 115 mg of NHS to activate one carboxylic acid of ThdCOOH. Then, this was magnetically stirred for 3 h, and 270 mg of PBAP was added. This reaction was continued for 12 h to synthesize ThdCOOH-PBAP conjugates. After that, 192 mg of EDAC and 115 mg of NHS were added to this reaction and then stirred for 6 h to activate another end of the carboxylic acid of the ThdCOOH-PBAP conjugates. Following this, 600 mg of COS dissolved in 10 mL of an H_2_O/DMSO mixture (1/4, v/v) was added to this reaction. Then, 24 h later, the reactants were introduced into the dialysis membranes and dialyzed against deionized water for 2 d to remove the organic solvent, unreacted chemicals, and byproducts. To avoid saturation, the deionized water was exchanged every 3 h intervals, and after that, the resulting solution was lyophilized to obtain solid products. The yield of the COSthPBAP conjugates was calculated as follows: Yield (%, w/w) = [(weight of ThdCOOH + weight of PBAP)/weight of COSthPBAP conjugates] × 100.

### 2.3. Characterization of COSthPBAP Conjugates

^1^H NMR spectra (500 mHz NB Fourier transform (FT)-NMR spectrometer, Varian Unity Inova; Varian Inc., Santa Clara, CA, USA) was employed to confirm the chemical composition and synthesis procedures of the conjugates. Each component and conjugates were dissolved in DMSO or a mixture of D_2_O/DMSO for analysis.

### 2.4. Fabrication of Ce6-Incorporated Nanophotosensitizers

COSthPBAP (40 mg) was reconstituted in 3 mL of deionized water, and then DMSO (5 mL) was added. To this solution, Ce6 dissolved in DMSO (2 mL) was added, and it was magnetically stirred for 10 min. This solution was poured into 10 mL of deionized water and introduced into a dialysis tube. The solution was dialyzed with deionized water over 1 d and the water was exchanged at 3 h intervals. The resulting solution was used for analysis or lyophilized for 2 d. For comparison, COSthPBAP nanoparticles were fabricated with a similar procedure without Ce6.

The Ce6 contents in the COSthPBAP nanoparticles were as follows: the lyophilized solids of the nanoparticles were distributed in deionized water (2 mL), and then DMSO (8 mL) was added. This solution was diluted with DMSO by more than 10 times, and then the Ce6 concentration was measured with a fluorescence spectrofluorophotometer (RF-5301PC, Kyoto, Japan). The excitation and emission wavelengths were 407 nm and 664 nm, respectively. The empty COSthPBAP nanoparticles were dissolved in H_2_O/DMSO (2/8 (v/v), 10 mL) and diluted with DMSO by ten times for a blank test.

Ce6 content (w/w, %) = (Ce6 weight/total weight of nanophotosensitizers) × 100.

Loading efficiency (w/w, %) = (Ce6 weight in the nanophotosensitizers/feeding weight of Ce6) × 100.

### 2.5. Characterization of Nanophotosensitizers

Transmission electron microscopy (TEM) (H7600, Hitachi Instruments Ltd., Tokyo, Japan) was used to observe the morphology of the nanophotosensitizers. The nanophotosensitizer solution 20 μL was dropped onto a carbon film-coated grid followed by drying at room temperature for 6 h. Phosphotungstic acid (0.1%, w/w in H_2_O) was used to negatively stain the nanophotosensitizers. The observation of the nanophotosensitizers was performed at 80 kV.

The ultraviolet-visible (UV) absorption spectrum of the nanophotosensitizers was analyzed with a Genesys 10s UV-VIS spectrophotometer (Thermo Fisher Scientific, Waltham, MA, USA).

A Zetasizer (Nano-ZS, Malvern, Worcestershire, UK) was used to measure the particle size of the nanophotosensitizers. For the effect of H_2_O_2_ on the particle size, the nanophotosensitizers fabricated as described above were diluted with phosphate-buffered saline (PBS, pH 7.4, 0.01 M), and H_2_O_2_ was added to this solution, followed by incubation at 37 °C for 3 h. This solution was used to measure the particle size.

The fluorescence properties of the nanophotosensitizers were measured with the fluorescence spectrofluorophotometer (RF-5301PC, Kyoto, Japan) between 500 nm and 800 nm (excitation wavelength: 400 nm). Images of the fluorescence properties of nanophotosensitizers were observed with a Maestro 2 small animal imaging instrument (Cambridge Research and Instrumentation Inc., Woburn, MA, USA). The nanophotosensitizers were reconstituted in phosphate-buffered saline (PBS, pH 7.4, 0.01 M), and H_2_O_2_ was added to this solution, followed by incubation at 37 °C for 3 h.

### 2.6. Drug Release Study

For the release study of Ce6, the nanophotosensitizer solution fabricated as described above was adjusted to 40 mL (1 mg/mg as a polymer) with deionized water, and then 5 mL of this solution was put into a dialysis membrane (MWCO: 2000 Da). After that, the dialysis tube was put into a conical tube with 45 mL of PBS (pH 7.4, 0.01 M). H_2_O_2_ was added to this solution to study the oxidative stress on the drug release rate from the nanophotosensitizers. The solution was incubated under shaking (100 rpm) at 37 °C. At predetermined time intervals, whole PBS was collected to measure the Ce6 concentration and then replaced with fresh PBS. The Ce6 concentration was measured with a fluorescence spectrofluorophotometer (RF-5301PC spectrofluorophotometer, Kyoto, Japan) at an excitation wavelength of 407 nm and emission wavelength of 664 nm. All results are the mean ± standard deviation (S.D.) from three separate experiments.

### 2.7. Devices for Light Irradiation

For the singlet oxygen (SO) generation and PDT study, an expanded homogenous beam (SH Systems, Gwangju, Korea) was used as reported previously [42]. The cells or nanophotosensitizers were irradiated at 664 nm and the light density was 2.0 J/cm^2^ [42]. The distance between the cells or nanophotosensitizer solutions from the LED panel was 40 cm. A 96-well plate or nanophotosensitizer solution was located in the center of the bottom panel. The light dose in the center of the bottom was determined with a photo-radiometer (DeltaOhm, Padova, Italy). The light density was measured at more than 20 points, and then the dose of light was calculated.

### 2.8. Singlet Oxygen (SO) Generation of Nanophotosensitizers

The generation of singlet oxygen (SO) generation by the Ce6 or nanophotosensitizers was evaluated as follows [43,44]: An aqueous solution (1 mL) of free Ce6 or nanophotosensitizers (5 µg/mL of Ce6 equivalent in distilled water, 1% DMSO) was made. SOSG reagent (final concentration: 5 µM) was added to this solution and then irradiated with an expanded homogenous beam (664 nm, SH Systems, Gwangju, Korea) at different time points (0.5, 1, 2, and 5 min). The fluorescence intensity was measured with a fluorescence spectrophotometer (RF-5301PC, Shimadzu Co., Kyoto, Japan) at the excitation wavelength of 488 nm and the emission wavelength of 525 nm. This measurement was performed under dark conditions.

### 2.9. Cell Culture and Culture Media

SCC-15 human tongue squamous cell carcinoma and HGF-1 human gingival fibroblast cells were purchased from the American Type Culture Collection (ATCC, Manassas, VA, USA). The SCC-15 and HGF-1 cells were maintained with Dulbecco’s modified eagle medium (DMEM)/F12, and DMEM media supplemented with 10% (v/v) fetal bovine serum (FBS) and 1% (v/v) antibiotics. YD-38 human gingival carcinoma cells and KB human oral cancer cells were purchased from the Korean Cell Line Bank Co. (Seoul, Korea). The cells were maintained with Roswell Park Memorial Institute (RPMI)-1640 medium supplemented with 10% (v/v) fetal bovine serum and 1% (v/v) antibiotics. The cells were cultured in a 5% CO_2_ incubator (37 °C). RPMI-1640 medium (Gibco^TM^, Ref. 11875-093) was purchased from Life Technologies Co. (Grand Island, NY, USA). DMEM medium (Gibco^TM^, Ref. 11995-065) was purchased from Life Technologies Co. (Grand Island, NY 14072, USA). DMEM/F-12 (Dulbecco’s modified Eagle’s medium/Nutrient mixture F-12, 1:1 mixture, Cat. LM 002-04) containing 4-(2-hydroxyethyl)-1-piperazineethanesulfonic acid (HEPES, 15 mM), L-glutamate (2.5 mM), and sodium bicarbonate (1200 mg/L) was purchased from Welgene Inc., (Gyeongsan-si, Gyeongsangbuk-do, Korea). FBS (Gibco^TM^, Ref. 12483-020) was purchased from Life Technologies Co. (Grand Island, NY, USA). According to the manufacturer’s data, the species for the FBS was cattle/bovine from Canada. Antibiotic solution (antibiotic–antimycotic (100×), Ref. 15240-062) was purchased from Life Technologies Co. (Grand Island, NY, USA). This solution was composed of streptomycin (10,000 µg/mL), amphotericin B (25 µg/mL), and penicillin (10,000 units/mL).

### 2.10. PDT of Oral Cancer Cells In Vitro

YD-38 or KB cells (2 × 10^4^ cells) seeded in 96-well plates were treated with Ce6 or nanophotosensitizers. The Ce6 was dissolved in DMSO for the Ce6 treatment and then diluted by more than 100 times with serum-free media (DMSO final concentration: 0.5% (v/v)). The nanophotosensitizers in deionized water were sterilized with a 1.2 µm syringe filter and then diluted with serum-free media. The cells were incubated for 2 h in a 5% CO_2_ incubator at 37 °C and, after that, washed with PBS twice. Serum-free fresh media (100 µL) were added to this, and then the cells were irradiated at 664 nm using an expanded homogenous beam (SH Systems, Gwangju, Korea). The light dose was 2.0 J/cm^2^. The light intensity was measured with a photo radiometer (Delta Ohm, Padua, Italy). The cells were further incubated for 24 h in a CO_2_ incubator at 37 °C. The viability of the cells was measured with an MTT (Sigma Aldrich Chem. Co., St. Louis, MO, USA) cell proliferation assay. MTT solution (30 µL, 5 mg/mL in PBS) was added to the 96 wells and then further incubated for 4 h in a CO_2_ incubator. The supernatants were removed and then replaced with 100 µL of DMSO. The viability of the cells was measured by absorbance at 570 nm using an Infinite M200 PRO microplate reader. The cell cultures and PDT procedures were carried out in dark conditions.

An intrinsic dark toxicity test was performed without the irradiation of light at 664 nm.

### 2.11. Intracellular Ce6 Uptake and ROS Generation of Oral Cancer Cells In Vitro

Cells (2 × 10^4^ cells) seeded into 96-well plates were treated with Ce6 or nanophotosensitizers for 2 h as described above and then washed with PBS twice. These cells were lysed with 50 µL of lysis buffer (GenDEPOT, Barker, TX, USA). The intracellular Ce6 uptake ratio was measured with the relative fluorescence intensity with an Infinite M200pro microplate reader (Tecan) (excitation wavelength: 407 nm, emission wavelength: 664 nm).

The intracellular ROS generation by the treatment of the Ce6 or nanophotosensitizers was evaluated with DCFH-DA. The cells (2 × 10^4^ cells) were treated with Ce6 or nanophotosensitizers in serum-free RPMI media with DCFH-DA (final concentration: 20 µm) for 2 h and then washed with PBS twice. Fresh phenol red-free RPMI media (100 µL) were added, and then the cells were irradiated at 664 nm (2.0 J/cm^2^). The intracellular ROS generation was measured at an excitation wavelength of 485 nm and an emission wavelength of 535 nm using a microplate reader (Infinite M200 PRO (Tecan)).

Fluorescence observation of the cells was carried out as follows: The cells (2 × 10^5^) were seeded on the cover glass in six-well plates and then treated with Ce6 or nanophotosensitizers for 1 h. After that, the cells were washed with PBS twice, fixed with 4% paraformaldehyde (PFA) solution in PBS for 15 m, immobilized with immobilization solution (Immunomount, Thermo Electron Co. Pittsburgh, PA, USA), and then observed with a fluorescence microscope (Eclipse 80i; Nikon, Tokyo, Japan).

### 2.12. Animal Tumor Imaging and In Vivo PDT Efficacy against AT84 Tumor Xenograft Model

For animal tumor imaging, KB cells (1 × 10^6^ cells) were subcutaneously injected into the backs of nude BALb/C mice (male, 20 g, five weeks old, OrientBio Co. Ltd. Seongnam-si, Gyeonggido, Korea). The KB cell-bearing mice were used for fluorescence imaging when the diameter of the tumor xenograft became larger than 6 mm. Nanophotosensitizer solution (10 mg Ce6/kg) was intravenously (i.v.) injected via the tail veins of the mice (injection volume: 200 µL). The whole bodies of the mice were observed with a Maestro^TM^ 2 small animal imaging instrument (Cambridge Research and Instruments, Inc. Woburn, MA, USA). For the fluorescence imaging of the tumors, the mice were anesthetized with avertin for whole-body imaging, and the organs were extracted to observe the organ distribution of the COSthPBAP nanophotosensitizers.

For the PDT of the mice, 0.5 mL of a stock solution of avertin (2,2,2-tribromoethanol, Sigma Aldrich Chem. Co. (St. Louis, MO, USA)) (25 g avertin in 15.5 mL tert-amyl alcohol) was mixed with 39.5 mL of 0.9% saline solution. The avertin solution (300~400 µL/mice) was intraperitoneally (i.p.) administered to anesthetize the mice.

According to the Ethical Program of the Pusan National University Institutional Animal Care and Use Committee (PNUIACUC), the mice were treated with CO_2_ (20%) and then the fluorescence images of the whole body and each organ were observed. The organs were extracted from the mice bodies and then fluorescence images were observed using a Maestro^TM^ 2 small animal imaging instrument (Cambridge Research and Instruments, Inc. Woburn, MA, USA). For the anticancer PDT treatment, the mice were anesthetized with the avertin solution and then irradiated with PDT equipment. Water and feed were freely provided to the mice. The mice were kept in the cage (3~4 mice/cage, Cage size: 200 mm × 260 mm × 130 mm (W × D × H)).

### 2.13. Statistical Analysis

The statistical significance of the results was evaluated by Student’s *t*-test using SigmaPlot^®^ (SigmaPlot^®^ v.11.0, Systat Software, Inc., San Jose, CA, USA), and *p* < 0.05 was evaluated as the minimal level of significance.

## 3. Results

### 3.1. Synthesis and Characterization of COSthPBAP Copolymer

**Figure 1:**
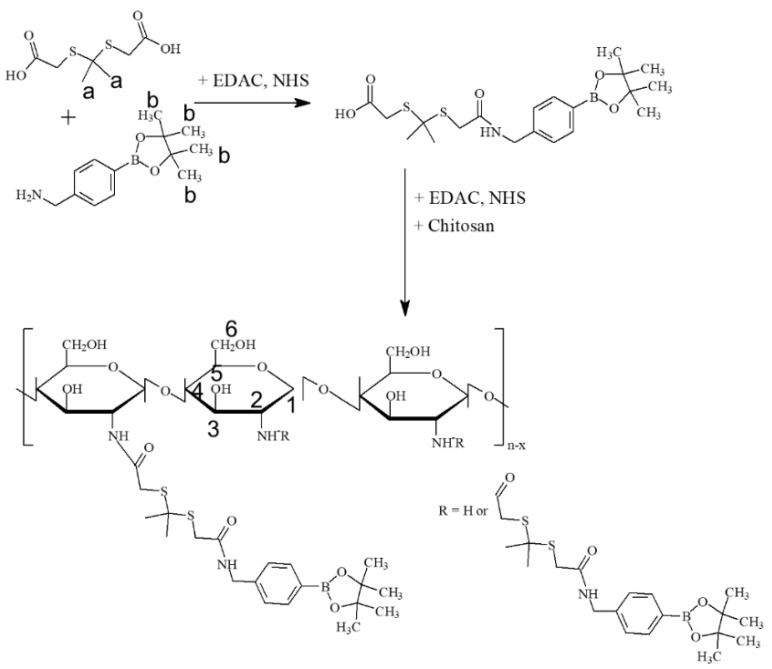
Synthesis scheme of COSthPBAP conjugates. “a” indicates the methyl groups of the ThdCOOH; “b” indicates the methyl groups of the PBAP. The numbers of “1–6” indicate te protons of carbon 1–6 of glucose in COS.

To make ROS-sensitive nanophotosensitizers, COSthPBAP conjugates were synthesized as shown in Figure 1. As shown in in Figure 1, one end of the carboxylic acid of ThdCOOH was activated with an EDAC/NHS system and then conjugated with PBAP to produce ThdCOOH-PBAP conjugates. Each specific peak of the methyl groups of the ThdCOOH (“a” in Figure 1) and PBAP (“b” in Figure 1) was confirmed at around 1.5~1.6 ppm and 1.3 ppm, respectively (Figure S1a,b). Specific peaks of glucosamine of COS were between 2.0 ppm and 5.0 ppm (Figure S1c), i.e., protons of carbon 1~6 (“1~6” of Figure 1) was confirmed. Another carboxylic acid end of the ThdCOOH-PBAP conjugates was activated with EDAC/NHS again and then conjugated with COS to produce COSthPBAP conjugates, as shown in Figure 1. The chemical structure and ^1^H NMR spectra of the COSthPBAP are shown in Figure S2a,b. As shown in Figure S2b, each specific proton peak of the COS, ThdCOOH, and PBAP was confirmed around 1.0 ppm~5.0 ppm, i.e., the specific peaks of the methyl protons of ThdCOOH and PBAP appeared at 1.8~1.9 ppm and 1.4~1.5 ppm, respectively. The peaks of COS also appeared at around 2.5~5.0 ppm. These results indicate that the COSthPBAP conjugates were successfully synthesized. The yield of the COSthPBAP conjugates was higher than 92.3% (w/w).

### 3.2. Characterization of Ce6-Incorporated COSthPBAP Nanophotosensitizers

Ce6-incorporated COSthPBAP nanophotosensitizers were prepared by the nanoprecipitation and dialysis method. The characteristics of the Ce6-incorporated COSthPBAP nanophotosensitizers were abbreviated as shown in Table 1. Higher Ce6 contents induced increases in the particle sizes of the nanophotosensitizers. Furthermore, the particle sizes were significantly increased compared to the empty nanoparticles. The Ce6 contents increased with increases in the feeding weight, but the loading efficiency was slightly decreased, as shown in Table 1.

**Figure 2:**
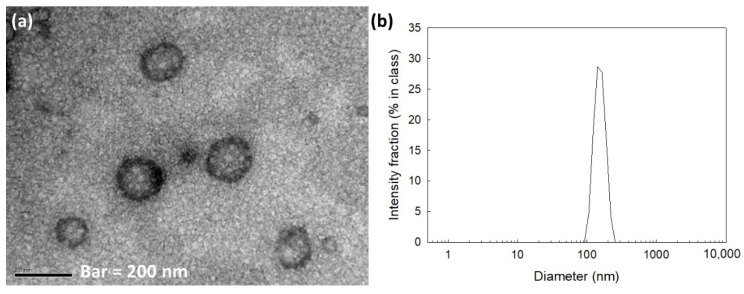
Morphological observations by TEM (**a**) and particle size distribution (**b**) of COSthPBAP nanoparticles (empty nanoparticles, 40/4 in Table 1).

As shown in Figure 2a, the COSthPBAP nanoparticles had nano-spherical shapes and small diameters of less than 200 nm. Their size distributions showed a monomodal distribution pattern (Figure 2b). Their average particle sizes were about 146 nm (Table 1).

**Figure 3:**
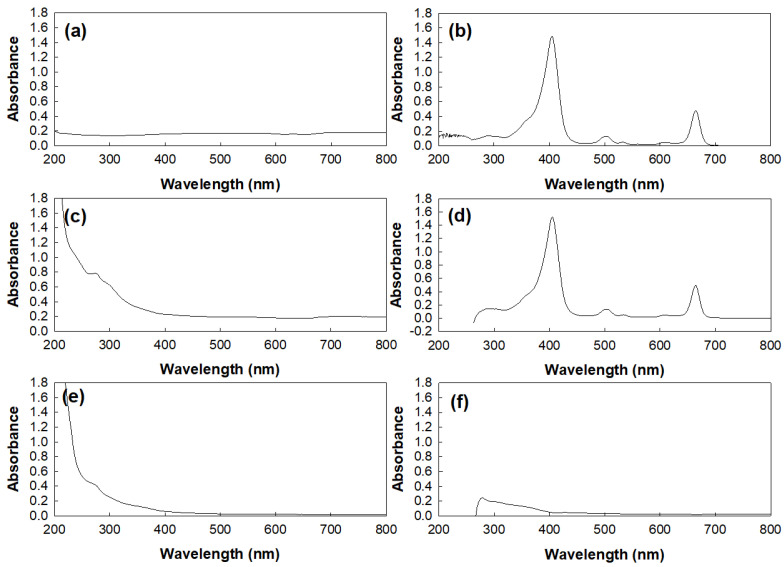
UV absorption spectra of Ce6, nanophotosensitizers, and empty nanophotosensitizers (COSthPBAP). (**a**) Ce6 (0.01 mg/mL) in deionized water (DW); (**b**) Ce6 (0.01 mg/mL) in DMSO; (**c**) nanophotosensitizers (0.01 mg/mL as a Ce6 concentration) in DW; (**d**) nanophotosensitizers (0.01 mg/mL as a Ce6 concentration) in DMSO-DW mixture (9:1, v:v); (**e**) empty nanoparticles (COSthPBAP, 1 mg/mL) in DW; (**f**) empty nanoparticles (COSthPBAP, 1 mg/mL) in DMSO-DW mixture (1:1, v:v).

Figure 3 shows the UV spectra of the Ce6, nanophotosensitizers, and empty nanoparticles in water (deionized water, DW), DMSO, and/or a DMSO-DW mixture. As shown in Figure 3a, no specific peaks of free Ce6 were observed in the DW because it had very low solubility, while it shows specific peaks between 300 nm and 700 nm in the DMSO (Figure 3b). The nanophotosensitizers did not have specific peaks higher than 400 nm, as shown in Figure 3c. When the nanophotosensitizers were dissolved in the DMSO-DW mixture, the UV spectra of the nanophotosensitizers show almost the same peak specificity as the free Ce6, as shown in Figure 3d. The empty nanoparticles had specific peaks lower than 400 nm in only the DW and the DMSO-DW mixture (Figure 3e,f). These results indicate that the COSthPBAP polymers did not affect the intrinsic properties of the free Ce6 during the nanophotosensitizer fabrication process.

**Figure 4:**
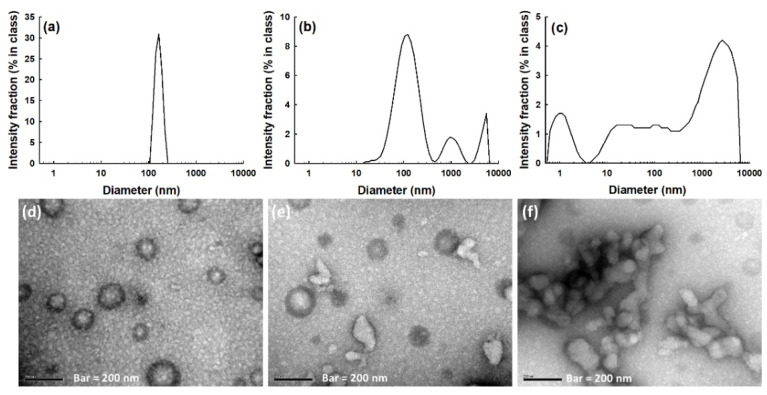
The effect of ROS on the changes in the particle size distribution (**a**–**c**) and morphology (**d**–**f**) of nanophotosensitizers. (**a**,**d**) H_2_O_2_, 0 mM; (**b**,**e**) H_2_O_2_, 0.5 mM; (**c**,**f**) H_2_O_2_, 2.0 mM. Nanophotosensitizers in PBS (pH 7.4, 0.01 M), incubated at 37 °C for 3 h in the absence or presence of H_2_O_2_.

To study whether or not the nanophotosensitizers had ROS sensitivity, the nanophotosensitizers were incubated in the presence of H_2_O_2_, as shown in Figure 4. The particle size distribution became multimodal and/or irregular (Figure 4b,c) in the presence of H_2_O_2_, i.e., the particle size distribution had a multimodal pattern with 0.5 mM of H_2_O_2_ and had an irregular distribution pattern with 2.0 mM of H_2_O_2_, while they maintained a monomodal distribution pattern with 0 mM of H_2_O_2_ (Figure 4a). The morphological observation with TEM also showed that the nanophotosensitizers were disintegrated in the presence of H_2_O_2_ (Figure 4e,f) while they maintained spherical shapes in the absence of H_2_O_2_ as shown in Figure 4d. These results indicate that the COSthPBAP nanophotosensitizers had ROS sensitivity and they were disintegrated by ROS.

**Figure 5:**
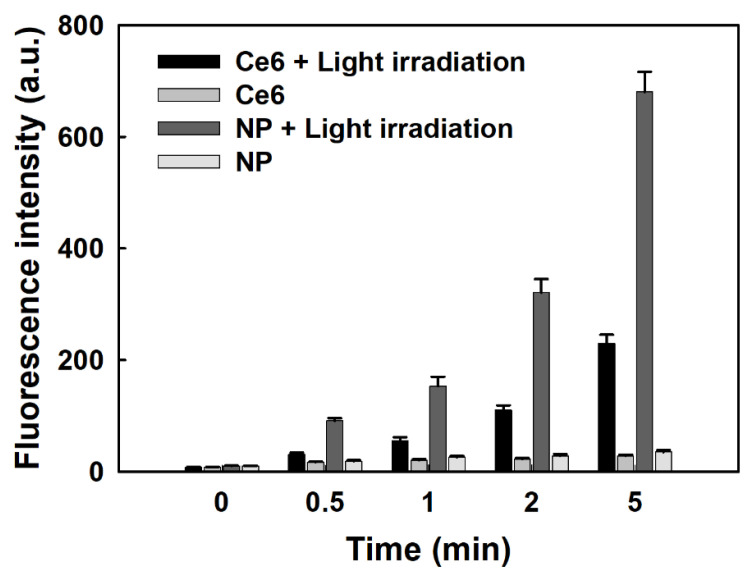
SO generation from free Ce6 and nanophotosensitizers in the absence or presence of light irradiation (664 nm, *n* = 4). Arbitrary units = (a.u.). NP= nanophotosensitizers. The results are the average ± standard deviation (s.d.) from three separate experiments.

Figure 5 shows the generation of SO from free Ce6 and nanophotosensitizers. As shown in Figure 5, the fluorescence intensity time-dependently increased both the free Ce6 and nanophotosensitizers under light irradiation, while changes in the fluorescence intensity were negligible in the absence of light irradiation. In addition, the fluorescence intensity of the nanophotosensitizers with light irradiation was more than two times higher than that of Ce6, indicating that the nanophotosensitizers efficiently produced ROS in the aqueous environment.

**Figure 6:**
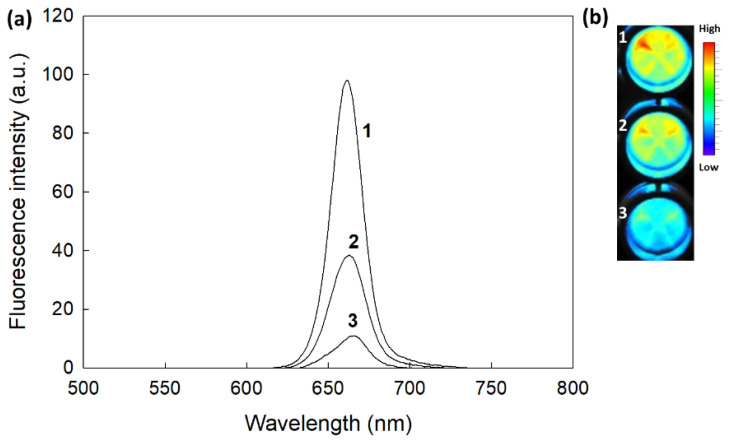
The effect of ROS on the changes in the fluorescence spectra (**a**) and fluorescence image (**b**) of nanophotosensitizers. Nanophotosensitizers were incubated for 3 h at 37 °C in the absence or presence of H_2_O_2_. 1. H_2_O_2_, 0 mM; 2. H_2_O_2_, 0.5 mM; 3. H_2_O_2_, 2.0 mM. The Ce6 concentration of the nanophotosensitizers in PBS was 0.05 mg/mL.

Figure 6 shows the changes in the fluorescence spectra and fluorescence images in the presence of H_2_O_2_. As shown in Figure 6a, the fluorescence intensity of the aqueous solution of the nanophotosensitizers gradually increased with H_2_O_2_, indicating that the nanophotosensitizers had ROS sensitivity and were able to respond to oxidative stress. Furthermore, fluorescence intensity in the images was also increased according to the H_2_O_2_ concentration (Figure 6b).

**Figure 7:**
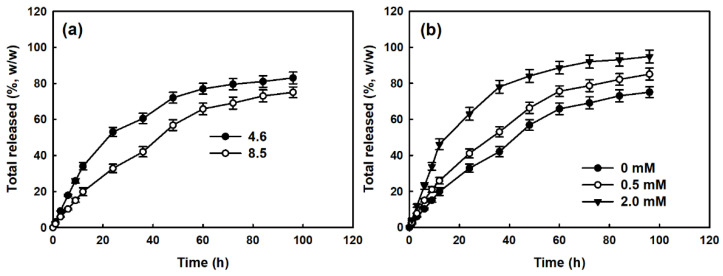
The effect of ROS on the Ce6 release from nanophotosensitizers. (**a**) The effect of Ce6 contents on the nanophotosensitizers. (**b**) The effect of H_2_O_2_ concentration on the Ce6 release from nanophotosensitizers. The results are the average ± standard deviation (s.d.) from three separate experiments.

Figure 7 shows the Ce6 released from the nanophotosensitizers. As shown in Figure 7a, the Ce6 release rate from the nanophotosensitizers was lower with a high Ce6 content, while the Ce6 release rate was faster with lower contents, indicating that the hydrophobic properties of Ce6 might have hydrophobically interacted in the core of the nanoparticles and then dissolved slowly. When H_2_O_2_ was added to the release media, the Ce6 release rate from the nanophotosensitizers was significantly faster with the H_2_O_2_ in a dose-dependent manner, as shown in Figure 7b. These results indicate that the Ce6 was released from the nanophotosensitizers in a ROS-sensitive manner.

### 3.3. Cell Culture Study In Vitro

**Figure 8:**
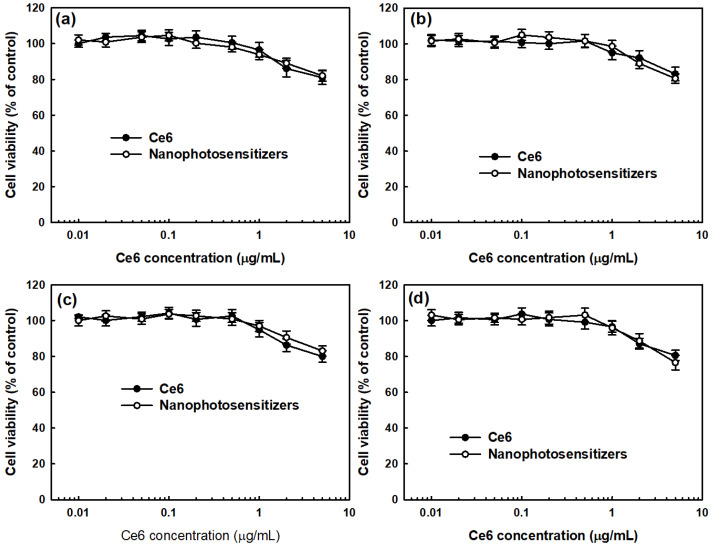
Dark toxicity of free Ce6 and nanophotosensitizers. (**a**) YD-38 cells. (**b**) KB cells. (**c**) SCC-15 cells. (**d**) HGF-1 cells. The results are the average ± standard deviation (s.d.) from eight different experiments.

Prior to testing the PDT efficacy against oral cancer cells, the intrinsic cytotoxicity of free Ce6 and nanophotosensitizers were evaluated as a means of dark toxicity (Figure 8). As shown in Figure 8a–c, both the Ce6 and nanophotosensitizers had low cytotoxicity until a 2 μg/mL Ce6 concentration against YD-38 cells, KB cells, and SCC-15 cells, i.e., the viabilities of YD-38 cells, KB cells, and SCC-15 cells were higher than 80% until a 2 μg/mL Ce6 concentration of the nanophotosensitizers and free Ce6. Furthermore, the nanophotosensitizers also had low intrinsic dark toxicity against HGF-1 human gingival fibroblast cells until 2 μg/mL of Ce6 and free Ce6 as shown in Figure 8d. These results indicate that the nanophotosensitizers and free Ce6 had low toxicity in the absence of light irradiation conditions. At 5 μg/mL, the free Ce6 and nanophotosensitizers resulted in less than 80% cell viability against AT84 cells and HGF-1 cells, respectively. The free Ce6 and nanophotosensitizers were tested until a 2 μg/mL Ce6 concentration for the next experiment. The results of the dark toxicity test indicate that the absence of light irradiation did not significantly affect the viability of the tumor cells or normal cells in either the free Ce6 or the nanophotosensitizers.

**Figure 9:**
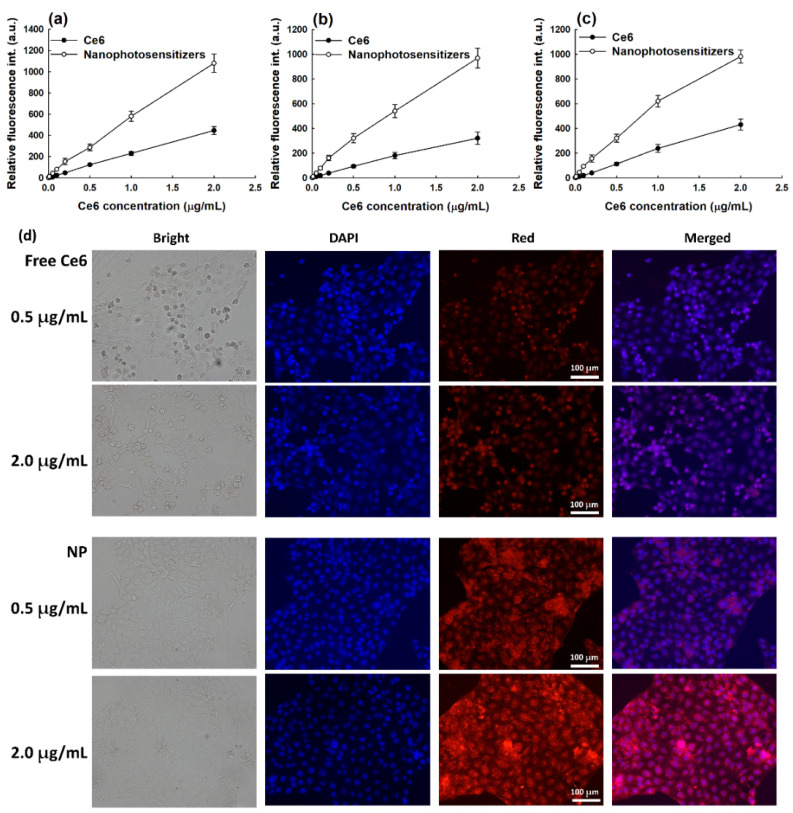
Intracellular Ce6 accumulation by treatment of Ce6 or nanophotosensitizers. (**a**) YD-38 cells. (**b**) KB cells. (**c**) SCC-15 cells. The results are the average ± standard deviation (s.d.) from eight different experiments. (**d**) Fluorescence observation of YD-38 cells after treatment of Ce6 and nanophotosensitizers. Relative fluorescence int. (a.u.) = Relative fluorescence intensity (arbitrary units). Bar = 100 μm. Magnification = 200×.

Figure 9 shows the relative Ce6 uptake ratio of the cancer cells. As shown in Figure 9a–c, the Ce6 uptake ratio dose-dependently increased in all cancer cells, including the YD-38 cells, KB cells, and SCC-15 cells, for both the free Ce6 and the nanophotosensitizers. Specifically, the Ce6 uptake ratio of the nanophotosensitizers was significantly higher than those of the free Ce6. These results indicate that the nanophotosensitizers had the superior potential for intracellular delivery. In the morphological observation of the YD-38 cells, the nanophotosensitizers revealed significantly stronger fluorescence intensity compared to treatment with the free Ce6 (Figure 9d). These results indicate that the nanophotosensitizers were internalized in the cells efficiently.

**Figure 10:**
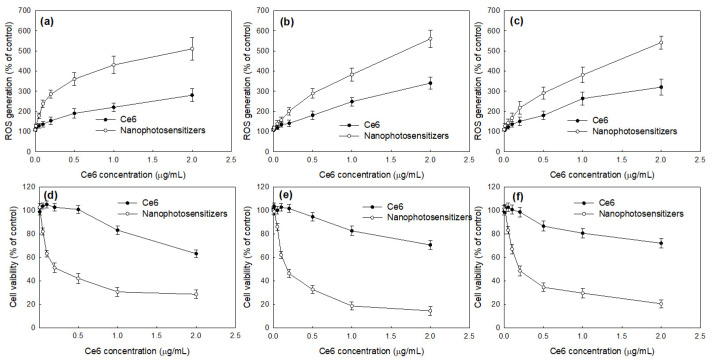
Relative ROS generation (**a**–**c**) and phototoxicity (**d**–**f**) by treatment of Ce6 or nanophotosensitizers. (**a**,**d**), YD-38 cells; (**b**,**e**), KB cells; (**c**,**f**) SCC-15 cells. The results are the average ± standard deviation (s.d.) from eight different experiments.

Figure 10 shows the relative ROS generation (Figure 10a–c) and PDT efficacy (Figure 10d–f) of the free Ce6 and nanophotosensitizers. As shown in Figure 10a–c, the relative ROS generation dose-dependently increased according to the Ce6 concentrations of both the free Ce6 and the nanophotosensitizers in all cancer cells, including the YD-38 cells (Figure 10a), KB cells (Figure 10b), and SCC-15 cells (Figure 10c). Furthermore, the nanophotosensitizers resulted in a higher ROS generation compared to the free Ce6, indicating that the nanophotosensitizers had a superior potential for generating intracellular ROS in cancer cells. Figure 10d–f shows the PDT efficacy of the free Ce6 and nanophotosensitizers against the YD-38 cells (Figure 10d), KB cells (Figure 10e), and SCC-15 cells (Figure 10f). As expected, the nanophotosensitizers showed higher phototoxicity against the YD-38 cells, KB cells, and SCC-15 cells compared to the free Ce6. These results indicate that the nanophotosensitizers had a higher potential for cellular uptake, ROS generation, and PDT efficacy compared to the free Ce6.

### 3.4. Animal Tumor Imaging of Tumor Xenograft Model

To investigate whether or not the nanophotosensitizers could target tumors, KB cells were implanted into the backs of nude mice, and then nanophotosensitizers were intravenously (i.v.) administered through the tail veins of mice.

**Figure 11:**
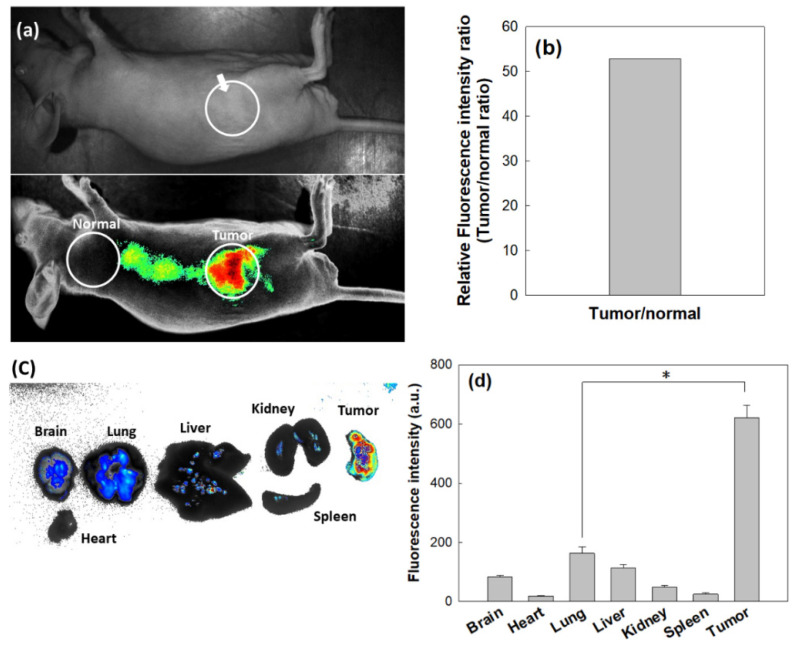
Animal tumor imaging using a KB cell-bearing tumor xenograft mouse model. (**a**) Fluorescence images of a whole mouse; (**b**) relative fluorescence intensity between tumor tissue vs. normal tissue; (**c**) fluorescence images of major organs; (**d**) fluorescence intensity of major organs. Nanophotosensitizer solution (10 mg Ce6/kg) was intravenously (i.v.) injected via the tail veins of the mice (Injection volume: 200 µL). The whole bodies of the mice were observed with a MaestroTM 2 small animal imaging instrument (Cambridge Research and Instruments, Inc. Woburn, MA, USA) after 24 h of i.v. administration. *, *p* < 0.01.

As shown in Figure 11a,b, strong fluorescence intensity in the tumor tissues was observed, i.e., red fluorescence was observed in the field of the tumor xenograft. As shown in Figure 11c,d, the fluorescence images of each organ show that the tumor revealed the strongest fluorescence intensity compared to the other organs. These results indicate that the nanophotosensitizers had the ability to target the tumors and then efficiently accumulated in the tumor tissues. This result may indicate that oral cancer can be imaged by the administration of COSthPBAP nanophotosensitizers and then efficiently cured by PDT.

## 4. Discussion

The traditional treatment regimen for oral cancer is currently considered to have limited efficacy due to delays in diagnosis and treatment [3,45]. Lauritzen et al. reviewed that delays in diagnosis have a significant correlation with the progression of the tumor stages, and the time from diagnosis to the treatment of oral cancer is significantly related to the survival of oral cancer patients [3]. Specifically, almost all cancers in the oral cavity and oropharynx are typically squamous cell carcinomas, which are flat and thin cells [1]. These cell types form the lining of the oral cavity, and these properties of oral cancers make it difficult to deliver anticancer drugs or biological therapeutics to oral cancer tissues [46]. Although various therapeutic regimens, such as surgery, chemotherapy, radiotherapy, and immunotherapy have been attempted to treat oral cancers, single and/or combined treatments are still problematic because high recurrence rates after these treatments have been reported, leading to a low survival rate of patients with oral cancers [47,48,49]. For these reasons, PDT can be considered a promising candidate for the treatment of oral cancers because PDT is suitable to be applied to squamous carcinoma types [50]. That is, the depth limit of light irradiation is known to be less than 2 mm, and thus, oral squamous carcinoma is suitable for light irradiation [17,51]. Furthermore, PDT using photosensitizers can be utilized to fluorescently detect and diagnose tumors in the oral cavity [52]. In addition, the unwanted side-effects of PDT, such as sun–shade problems, are always problematic in PDT approaches for cancers [28]. In a clinical application, talaporfin sodium-based PDT showed the safe regression of esophageal cancer against local failure after chemoradiotherapy [27].

Nanoparticle-based PDT, such as nanophotosensitizers, is believed to be a promising candidate for the tumor-specific delivery of photosensitizers [38,39,53]. Since nano-dimensional carriers can be accumulated in the tumor tissue via the enhanced permeation and retention (EPR) effect, nano-based medicine has been extensively investigated for cancer therapy [53,54,55]. Specifically, tumor microenvironments are quite different compared to normal tissues and cells [55,56]. The redox potential of tumor microenvironments is known to be elevated and can be applicable for tumor-targeting using nano-based medicine [57,58]. Mirhadi et al. reported that redox-sensitive nanomedicine can be used to target cancer cells and emphasize the anticancer activity of therapeutics [58]. In our results, COSthPBAP nanophotosensitizers released Ce6 in an ROS-sensitive manner through the ROS-sensitive disintegration of the COSthPBAP nanophotosensitizers, as shown in Figure 4, Figure 6 and Figure 7. The COSthPBAP nanophotosensitizers responded to oxidative stress in the presence of H_2_O_2_ and then were efficiently delivered to cancer cells as free Ce6. These trends of nanophotosensitizers are related to the generation of ROS and PDT efficacy, i.e., the COSthPBAP nanophotosensitizers showed superior ROS generation and PDT efficacy against oral cancer cells with low dark toxicity against normal HGF-1 cells (Figure 8, Figure 9 and Figure 10). Photosensitizers, such as 5-amino levulinic acid (5-ALA), can be used to improve the diagnostic contrast/accuracy of oral cancers through the fluorescence detection of anatomic locations of the oral cavity [59]. PDT treatment against oral leukoplakia lesions and oral lichen planus lesions showed positive results [60]. However, the low tumor specificity of traditional photosensitizers frequently results in dispersion in normal cells or tissues, and these properties are related to the side effects of traditional photosensitizers. COSthPBAP nanophotosensitizers can be specifically delivered to tumor tissue, i.e., the fluorescence intensity was the strongest in the tumor tissues compared to other organs (Figure 11), indicating that COSthPBAP nanophotosensitizers can be delivered to tumor tissues specifically.

**Figure 12:**
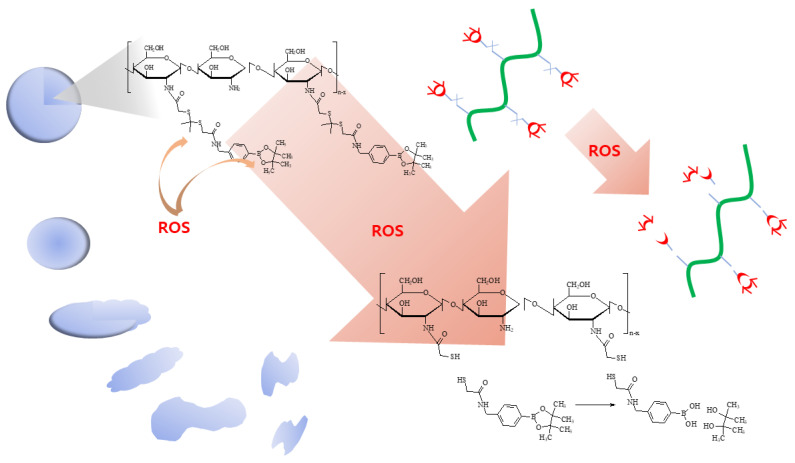
Schematic illustrations of Ce6-incorporated COSthPBAP nanophotosensitizers.

As illustrated in Figure 12, the degradation of the thioketal group and PBAP moiety is known to be affected by the presence of ROS [61,62,63]. Lee et al. also reported that phenyl boronic acid can be degraded in the presence of ROS, such as H_2_O_2_, and, after that, acid release by H_2_O_2_ catalyzes the hydrolysis of the polymer backbone [61]. These behaviors induce changes in the polymer properties from hydrophobic to hydrophilic. Gao and Xiong also showed that the thioketal group can be degraded by reactive oxygens and then degraded into thiol groups [62]. PBAP moieties and thioketal linkers in the polymer backbone resulted in hydrophobic–hydrophilic changes and degradation by ROS-sensitive behavior [63]. These changes accelerated the release rate of bioactive agents and anticancer drugs and then emphasized their anticancer activities. Our results also indicate that the drug release rate was accelerated by H_2_O_2_ and then affected the ROS production/PDT effect, as shown in Figure 6, Figure 7, Figure 8, Figure 9 and Figure 10. 

Nanomaterials, polymer conjugates, and/or nanoparticles are known to accelerate the generation of singlet oxygen rather than free Ce6 [44,64,65]. For example, Park and Na reported the measurement of Ce6-pluronic F127 conjugates generating singlet oxygen using SOSG reagent was significantly better compared to free Ce6 [44]. They argued that the singlet oxygen generation of Ce6-pluronic F127 conjugates was five times higher than that of free Ce6 due to the improved aqueous solubility against distilled water. Nanomaterials, such as carbon nanotubes, are also reported to enhance singlet oxygen generation [64]. Nanoparticles based on polymers can be considered an ideal vehicle to improve aqueous solubility, photostability, and photo dynamic activity [65]. Our results also indicate that the COSthPBAP nanophotosensitizers resulted in higher singlet oxygen generation than free Ce6 (Figure 5).

## 5. Conclusions

COSthPBAP copolymers were synthesized for Ce6 delivery against oral cancer cells. ThdCOOH was conjugated with PBAP to produce ThdCOOH-PBAP conjugates and then added to the amine groups of COS to produce COSthPBAP copolymers. Ce6-incorporated nanophotosensitizers using the COSthPBAP copolymers were fabricated using the nanoprecipitation and dialysis methods. The Ce6-incorporated COSthPBAP nanophotosensitizers had small diameters of less than 200 nm, with a mono-modal distribution pattern. However, they became multimodal and/or irregular distribution patterns when H_2_O_2_ was added. In the morphological observation using TEM, the nanophotosensitizers were disintegrated by the addition of H_2_O_2_, indicating that the COSthPBAP nanophotosensitizers had ROS sensitivity. In addition, the Ce6 release rate from the COSthPBAP nanophotosensitizers accelerated in the presence of H_2_O_2_. SO generation was higher in the nanophotosensitizers than in the free Ce6. Furthermore, the COSthPBAP nanophotosensitizers showed a higher intracellular Ce6 uptake ratio and ROS generation in all types of oral cancer cells. They also efficiently inhibited the viability of oral cancer cells under light irradiation, but they did not significantly affect the viability of either normal cells or cancer cells in the absence of light irradiation. The COSthPBAP nanophotosensitizers showed a tumor-specific delivery capacity and fluorescence imaging of KB tumors in an in vivo animal tumor imaging study. We suggest that COSthPBAP nanophotosensitizers are promising candidates for the imaging and treatment of oral cancers.

## Figures and Tables

**Table 1:** Characterization of COSthPBAP nanophotosensitizers.

COSthPBAP/Ce6 Weight (mg/mg)	Drug Contents (%, w/w) ^1^	Loading Efficiency (%, w/w) ^1^	Particle Size (nm) ^2^	Polydispersity	Zeta Potential (mV) ^3^
40/0	-	-	84.4 ± 4.5	0.093	8.2 ± 0.35
40/2	4.6	96.0	132.9 ± 5.2	0.111	4.6 ± 0.19
40/4	8.5	93.0	146.2 ± 4.1	0.107	3.1 ± 0.23

^1^ Drug content (w/w, %) = (Ce6 weight/total weight of nanophotosensitizers) × 100.; Loading efficiency (w/w, %) = (Ce6 weight in the nanophotosensitizers/feeding weight of Ce6) × 100. ^2^ Particle sizes were average ± standard deviation from three different measurements. ^3^ Zeta potential was average ± standard deviation from three different measurements.

## Data Availability

Not applicable.

## Supplementary Materials

The following supporting information can be downloaded at: https://www.mdpi.com/article/10.3390/ma15207057/s1, Figure S1: Chemical structure and 1H NMR spectra. (a) ThdCOOH; (b) PBAP; (c) COS; Figure S2: Chemical structure (a) and 1H NMR spectra (b) of COSthPBAP conjugates.
