# Supplementary material for: Nanophotosensitizers Composed of Phenyl Boronic Acid Pinacol Ester-Conjugated Chitosan Oligosaccharide via Thioketal Linker for Reactive Oxygen Species-Sensitive Delivery of Chlorin e6 against Oral Cancer Cells
Source: Materials (Basel). 2022 Oct 11;15(20):7057. doi: 10.3390/ma15207057 (PMC9604738; doi:10.3390/ma15207057)
Supplement: Supplementary file 1 [file materials-15-07057-s001.zip › materials-1874396-supplementary.pdf]

Supplementary Materials

Experimental

Characterization of chemicals and conjugates

^1H NMR spectra (500 mHz NB Fourier transform (FT)-NMR spectrometer, Varian Unity Inova; Varian Inc., Santa Clara, CA, USA) was employed to confirm chemical composition and synthesis procedures of conjugates. Each component and conjugates were dissolved in DMSO or mixtures of D_2O/DMSO for analysis.

Results

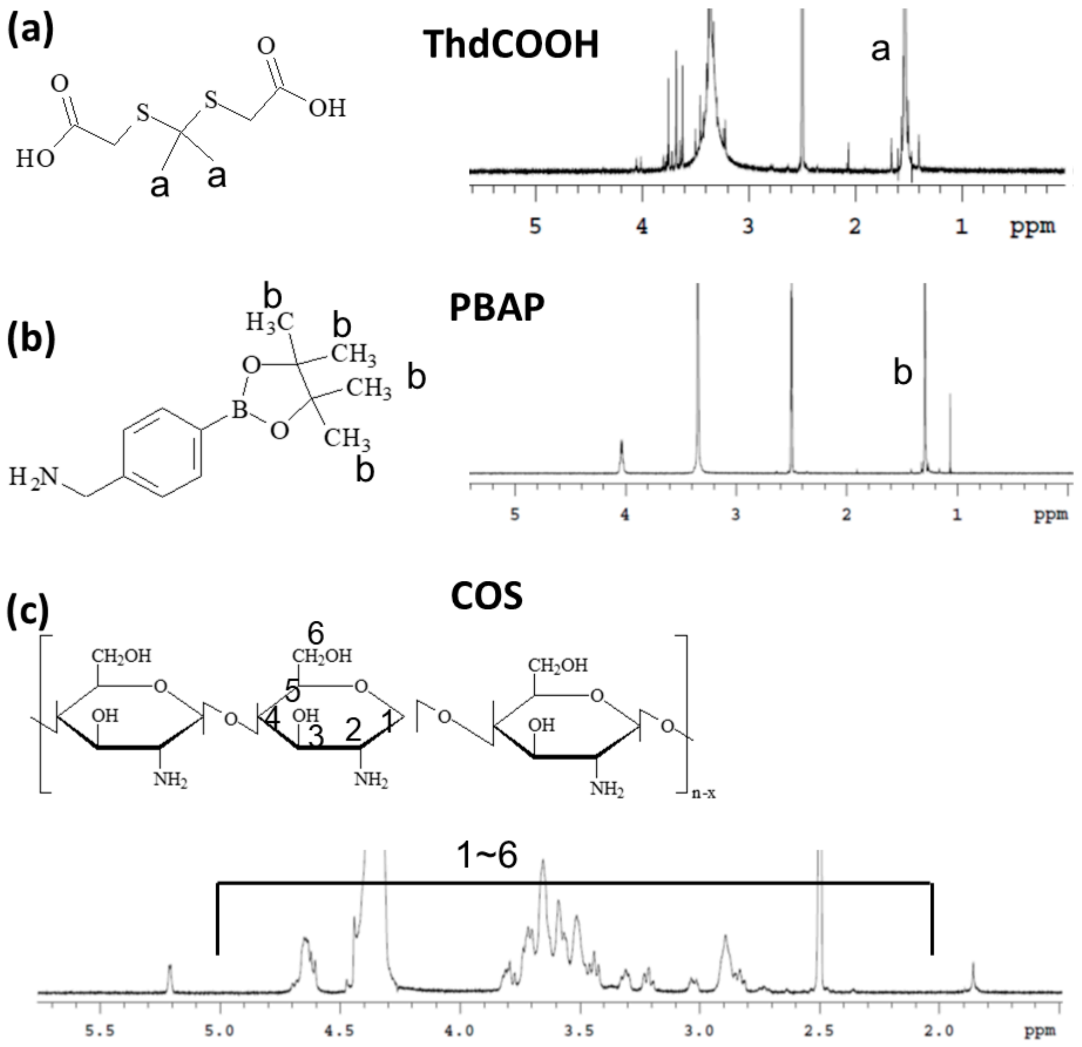

Figure S1. Chemical structure and ^1H NMR spectra. (**a**) ThdCOOH; (**b**) PBAP; (**c**) COS.

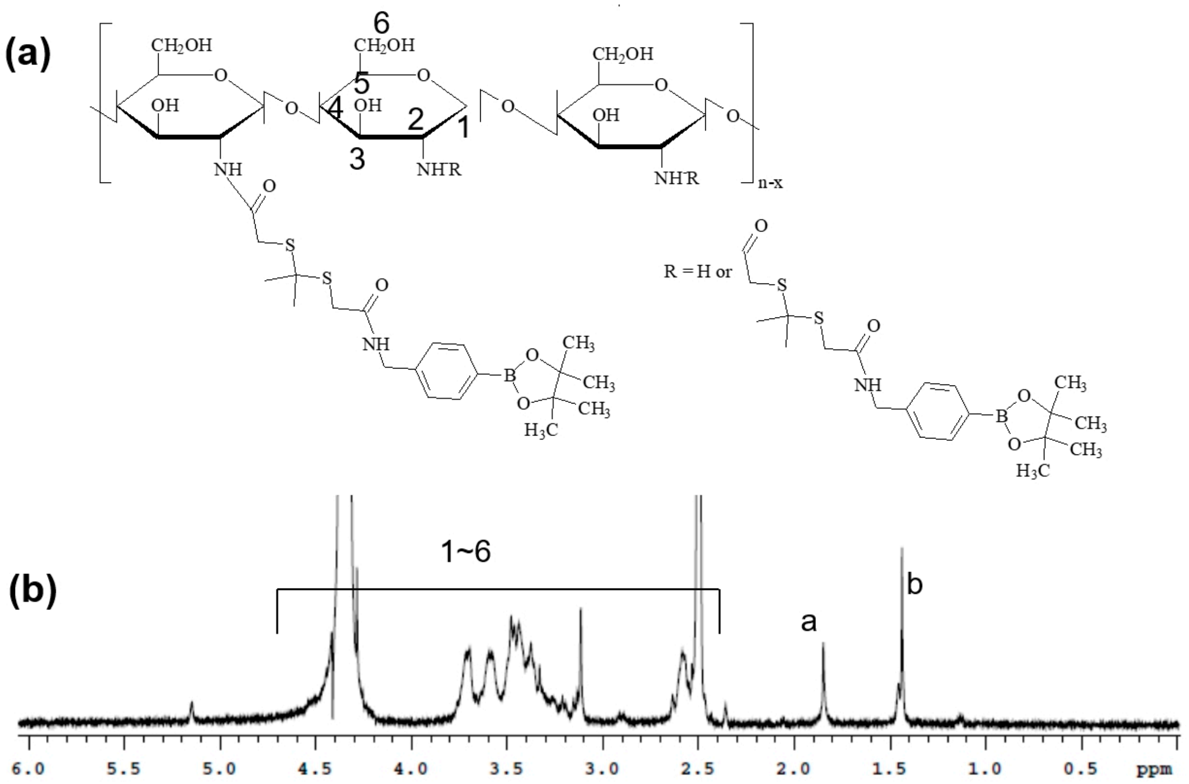

Figure S2. Chemical structure (a) and ^{1}H NMR spectra (b) of COSthPBAP conjugates.
